# A “Noodle and Thread”: A Low-Fidelity Simulation of Blood Vessel Ligation Using Common Household Items

**DOI:** 10.1007/s40670-025-02315-w

**Published:** 2025-02-20

**Authors:** Justin Markel, Jacob D. Franke, Kerri Woodberry, Matthew Fahrenkopf

**Affiliations:** 1 Cedars-Sinai Medical Center Department of Internal Medicine, West Hollywood, USA; 2 Corewell Health-Michigan State University College of Human Medicine Plastic Surgery Residency, Grand Rapids, MI USA; 3 Department of Plastic and Reconstructive Surgery, West Virginia University School of Medicine, Morgantown, USA; 4 Elite Plastic Surgery Group, Grand Rapids, USA

**Keywords:** Vessel ligation, Surgical education, Noodles, Blood vessel, Simulation

## Abstract

**Purpose:**

Simulation is becoming increasingly essential to surgical education, and many skills are learned in simulation-based training laboratories before being used in the operating room (OR). The COVID-19 pandemic exposed the need for alternative methods of learning important surgical techniques, particularly in resource-limited areas. One of the most important early skills for trainees is surgical knot tying without exerting excessive upward traction, such as that used to ligate blood vessels prior to division and cautery.

**Methods:**

To help solve this problem, we have developed a model of blood vessel ligation and surgical knot tying using common, inexpensive household items including noodles, adhesive tape, and thread.

**Results:**

The proposed model adequately simulated blood vessel ligation and displayed a wide spectrum of difficulty levels based on the materials chosen.

**Conclusions:**

Surgical knot tying can be practiced in private settings with the proposed model of blood vessel ligation. The model is low cost, and its difficulty can be adjusted by changing noodle morphology.

## Introduction

Aspiring surgeons develop their skills at variable rates. Techniques such as knot tying, suturing, and blood vessel ligation are essential across all surgical specialties; however, it can be challenging for students to practice surgical techniques outside of the operating room (OR), especially in resource-limited areas. As technologies develop, many resource-abundant programs are shifting towards a simulation-based approach to surgical education, which allows for unlimited practice in a low-stakes environment [1, 2]. However, as simulations become more advanced, their price tags increase concomitantly, which may limit their utility in resource-limited regions [3]. Indeed, surgical simulation systems, such as the BOSS Technical Skills Package, are currently listed at $895 (USD) as of January 2025 [4]. While effective, these tools may not be readily available for students and institutions with limited resources.

Low-cost simulation models can help address these disparities by providing affordable educational opportunities for learners regardless of geographic or socioeconomic background. It is well-recognized that low-cost simulations have the potential to provide substantial training value with the added benefit of being more easily accessible to students [5]. For instance, Walsh et al. (2016) proposed a model of auricular hematoma repair using bell peppers, cardboard, plastic wrap, and ketchup [6]. In addition, as highlighted by the recent COVID pandemic, it is becoming especially useful to have simple yet effective ways to practice surgical techniques from one’s own living space.

One of the most important basic surgical techniques is surgical knot tying, and trainees must master the ability to tie one-handed and two-handed surgical knots in a timely fashion. In addition, they must develop the skill of tying secure knots without exerting excessive upward traction, which becomes particularly important when tying knots against fragilis tissues prone to avulsion, such as bleed vessels. Currently, there are a wide variety of simulators available that provide realistic experiences and improve technique; however, many are costly and require special equipment, storage space, and access to animals and cadavers [7, 8]. Substantially lower-cost simulation systems have been shown to provide an effective training experience [9]. In 2012, Dastur published a do-it-yourself method to practice blood vessel suturing using an empty 330-ml aluminum can [10]. This model, along with many others, In this manuscript, we describe a novel method for practicing blood vessel ligation and knot tying using other common and inexpensive household items. The current model has the potential to make surgical training tools more accessible to learners both within the USA and abroad.

## Materials and Methods

Noodles are a common food used in a variety of dishes, made from a variety of ingredients (e.g., wheat, rice), and found in numerous cuisines across the globe [11]. They are inexpensive and come in a variety of shapes and sizes, all of which can be used to model different blood vessel sizes and tortuosities [12]. Moreover, noodles share a variety of mechanical properties with blood vessels, such as tensile strength, elasticity, and viscoelasticity, which can be adjusted depending on their exposure to water, salt, and heat [13–15]. Blood vessels receive their elastic properties from varying amounts of collagen and elastin proteins in their walls. Veins are low-resistance conduits with thinner, weaker walls, while arteries are exposed to higher hydrostatic forces and stronger-walled [14, 16]. Raw or minimally cooked noodles mimic the stiffness of calcified vessels, while *al dente* noodles more closely mimic the mechanical properties of uncalcified vessels. Indeed, studies have described the tendency of noodles to deform and undergo mechanical failure when various forces are applied; Brookfield Ametek found that an average of 8 ± 0.7 g of force was required to break a spaghetti noodle cooked in 300 ml of boiling water and cooled for 1 min prior to testing. Previous articles have also recognized the utility of noodles for surgical skills training and described the use of hollow noodles to model blood vessel anastomosis [9] [17].

**Fig. 1:**
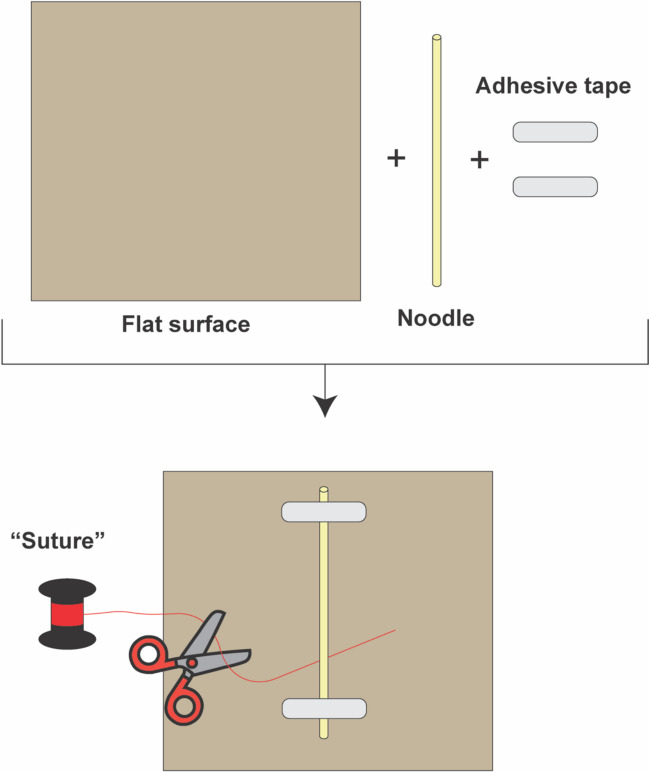
Schematic depicting setup for modeling blood vessel ligation using noodles. The noodle is first secured to a flat surface using adhesive tape. Next, the “suture,” which in this case is thread, is fed under the noodle and tied

To set up this simulation, only three materials are required: (1) noodles, (2) suture material, and (3) tape (or some other adhesive substance). To construct the model, a noodle of the desired diameter and shape is taped to a flat surface at opposite ends, leaving a few centimeters in between to allow for deformation of the noodle with upward traction. Suture is then fed under the noddle at a point equidistant from the anchor points and hand or instrument tied using proper surgical technique (Fig. 1).

Any type of thread or string material can be substituted for suture. Dental floss is another suitable alternative if string or thread is unavailable. When tying around the noodles, care is taken to not disrupt their physical integrity. The model becomes increasingly challenging by soaking or boiling the noodles in water for increasing periods of time, as exposure to moisture and heat makes it more prone to tearing; this scenario is analogous to tying knots on smaller vessels or more friable tissues. The model provides immediate feedback to the trainee when excessive traction causes the noodle to tear. Cooking times can be extended to further challenge trainees as they progress. Additionally, as noodles come in all different shapes and sizes, the complexity of the model can be enhanced by choosing noodles with different morphologies. As an example, tying knots across the spiral-shaped Ramen® noodle can simulate ligation of a more tortuous vessel.

## Results and Discussion

Medical students and residents around the world may benefit from low-cost blood vessel ligation models that can be assembled cheaply and quickly and performed at home. While some trainees can afford to buy suture practice kits, those in more resource-limited regions within the USA and globally may be limited by cost. Further, there is often a limited amount of suture material included in each kit, and it is relatively expensive to replace. Herein, we describe a novel simulation model of blood vessel ligation using low-cost, common household products that provide modifiable levels of difficulty and variety.

Noodles are an extremely common food item around the globe. The total cost of the proposed model (i.e., noodle, tape, and thread) is around $10 US dollars (USD), and it can be purchased in the USA at gas stations, markets, and other stores selling sewing supplies and groceries. Moreover, each box of noodles and spool of thread have ample materials to conduct multiple simulations without the need to restock materials.

**Fig. 2:**
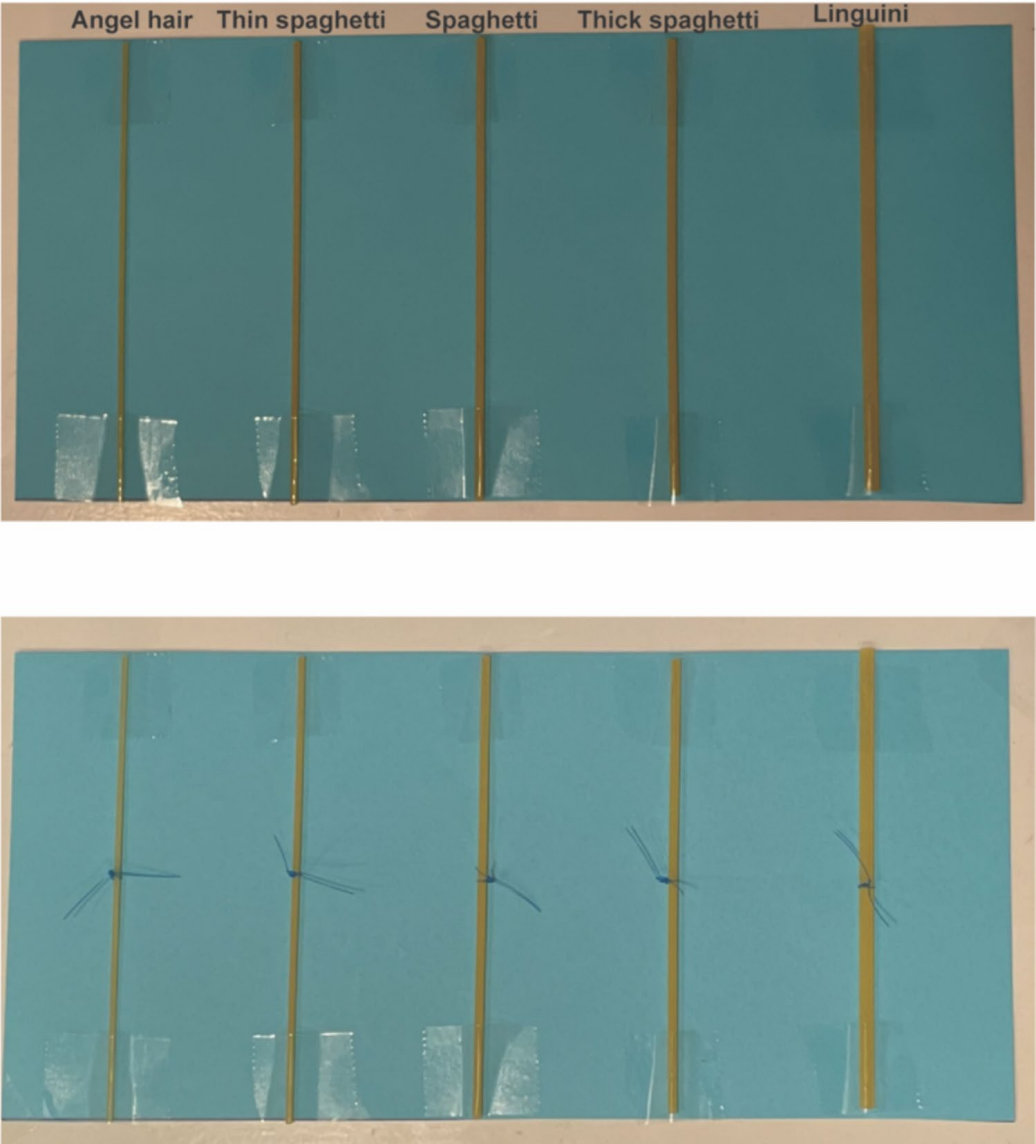
Common spaghettis with different diameters. Examples of five common Barilla® Italian spaghettis of various diameters taped with clear tape to a piece of construction paper for contrast, ordered left to right by increasing diameter (angel hair, thin spaghetti, spaghetti, thick spaghetti, and linguini) without (top) and with (bottom) example 3–0 nylon suture ties

**Fig. 3:**
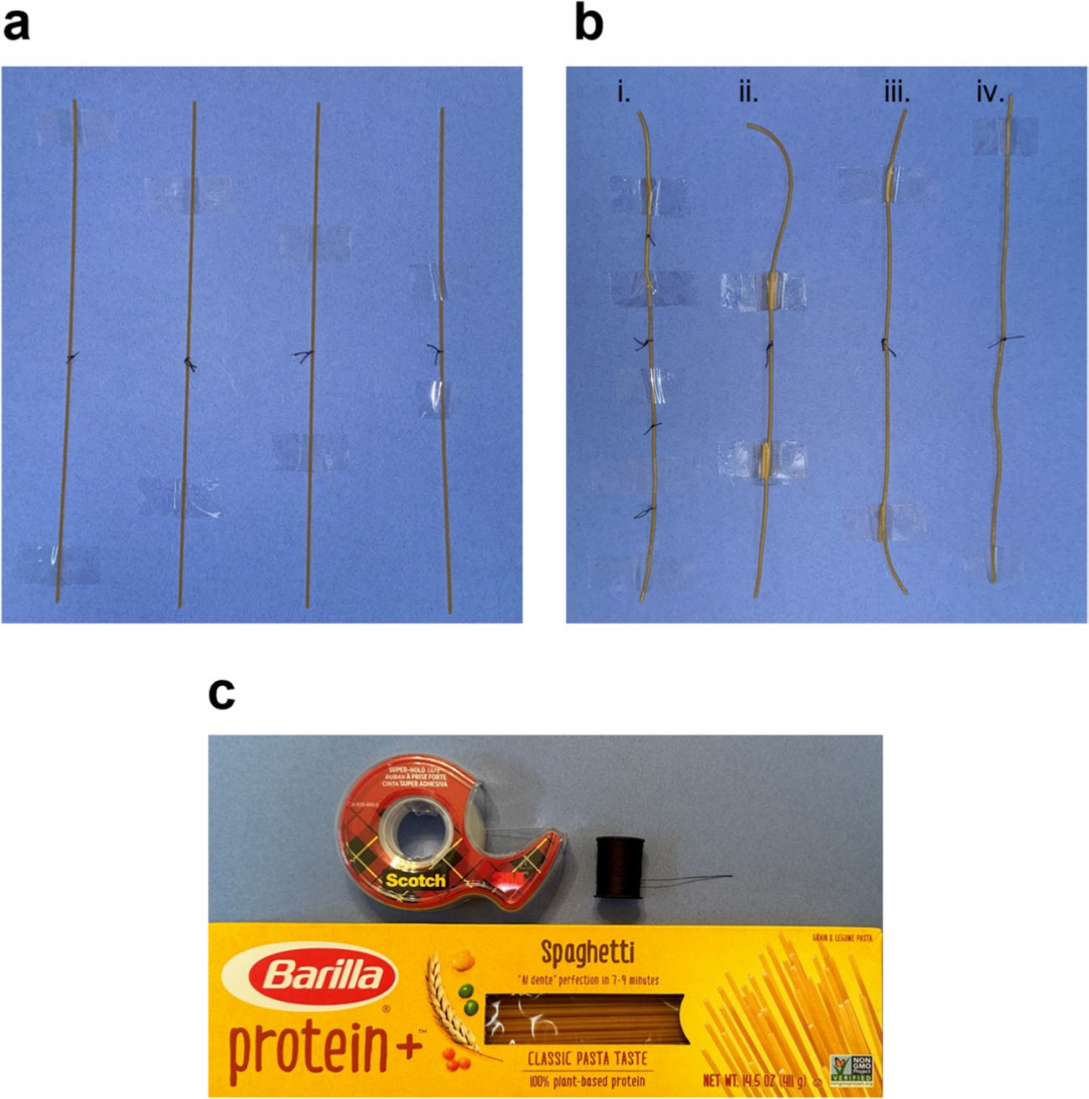
Depiction of Barilla® protein-enriched spaghetti tied with common black thread and secured with clear Scotch® tape. **a** Uncooked pasta with knots tied with black thread. **b** Cooked pasta with knots tied with black thread (after being cooked in boiling water for 4 min). Noodles ii–iv are secured only at their distal ends, while noodle i is secured at four points, allowing for more knots to be tied on the same noodle substrate. **c** Materials used to create the model depicted in this figure

Using noodles of varying diameters allows the trainee to practice tying vessels of various diameter and tensile strengths. In Fig. 2, we show five common types of Barilla® spaghetti of varying diameters found at most grocery stores across the USA with and without 3–0 nylon suture ties. In the diagram, the noodles are organized in order of increasing diameter from left to right and include angel hair, thin spaghetti, spaghetti, thick spaghetti, linguini, and tagliatelle. The thinner the noodle, the less traction is required to break it and hence the greater the challenge. The difficulty can be further modified by cooking the pasta. Figure 3 shows protein-enriched pasta tied with store-bought thread pre- and post-cooking for 4 min in boiling water (approximately 50% of the recommended cooking time). As depicted in Fig. 3B, the pasta can be taped at multiple locations to increase the number of knots that can be tied on the same noodle.

We suggest that trainees start by practicing one-handed and two-handed knot tying on uncooked noodles of thicker diameters. Trainees are encouraged to keep a record of the time needed to complete each knot to track their knot-tying efficiency. Next, if possible, trainees can vary the caliber of thread and thickness and pliability of the noodle. Moreover, wearing gloves while tying will better simulate the operative experience. As trainees advance, they can begin softening the noodles by soaking or boiling them in water to decrease their tensile strength and increase the challenge. As the noodle softens, the amount of upward tension required to rupture the noodle is decreased, and more skill is required to complete the knot with an intact noodle.

## Conclusions

Surgical knot tying and vessel ligation are two of the most important basic surgical skills for early surgical training. As simulation models advance and rise in cost, limitations availability and accessibility may preclude widespread use. Using the “noodle and thread” model of blood vessel ligation described in this article, students can practice in the comfort of their own homes and improve their skills in a safe, socially distanced, and low-stress environment. Further underscored by the recent COVID-19 pandemic, it is important to have alternative options for at-home learning for medical students and residents. This model may be useful for trainees around the globe, particularly those in resource-limited areas. We also hope to promote this model to trainees across the globe using popular social media platforms.
